# A Rare Case of *Granulicatella adiacens* Vertebral Osteomyelitis

**DOI:** 10.1155/2021/1483846

**Published:** 2021-12-13

**Authors:** E. van der Palen, C. L. M. de Roij van Zuijdewijn, D. A. R. Castelijn, G. H. Wattel-Louis, J. Kalpoe

**Affiliations:** ^1^Department of Internal Medicine, Spaarne Gasthuis Hospital, Haarlem, Hoofddorp, Netherlands; ^2^Department of Internal Medicine, Amsterdam University Medical Centre, Amsterdam, Netherlands; ^3^Regional Public Health Laboratory Kennemerland, Haarlem, Netherlands

## Abstract

Vertebral osteomyelitis caused by *Granulicatella adiacens* is rarely described. We report a 45-year-old immunocompetent male with back pain caused by *G. adiacens* osteomyelitis. This case is remarkable due to the absence of endocarditis. A clinician should therefore consider *G. adiacens* osteomyelitis even in the absence of concurrent hematogenous spread.

## 1. Case Report

**Figure 1:**
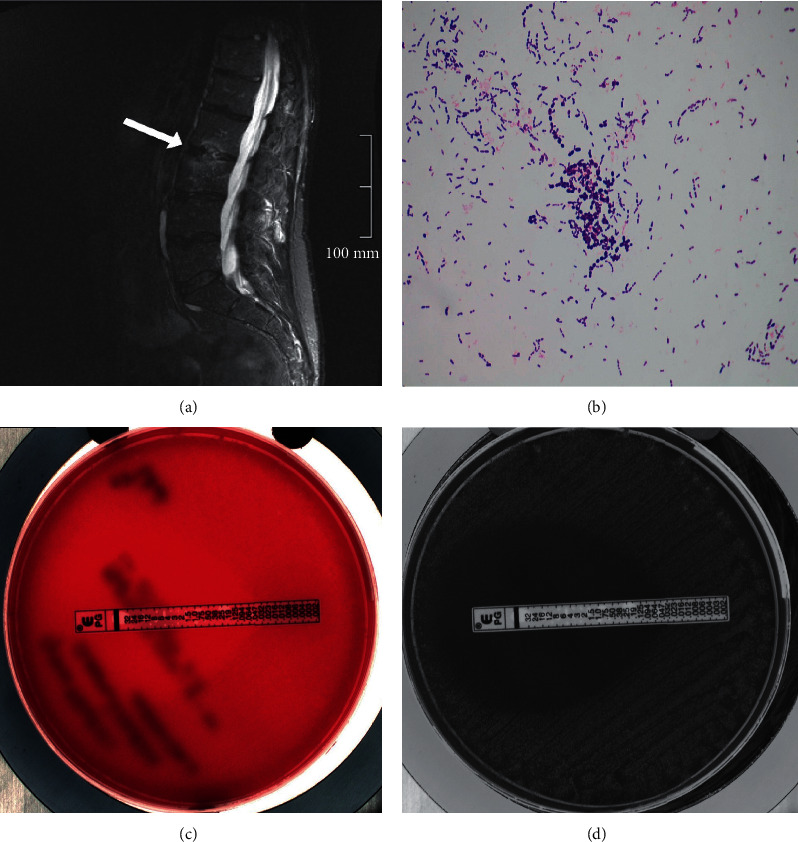
(a) MRI lumbar spine, T2-weighted image. (b) Gram stain of positive blood culture showing Gram-positive cocci. (c)-(d) Blood cultures and antibiotic susceptibility testing.

A 45-year-old Caucasian male visited his gastroenterologist for follow-up on Crohn's disease, which was limited to the terminal ileum. Three months prior to the present episode, treatment with 6-mercaptopurine was discontinued due to elicited leukopenia. There were no clinical signs of activity of Crohn's disease, but he reported an acute worsening of chronic lower back pain for three weeks, which was accompanied by chills for two days. Due to the absence of fever, he was discharged after blood cultures were drawn. After 48 hours, a Gram-positive coccus (*Granulicatella adiacens)* was detected, and he was requested to come to our emergency department. He reported no recent history of dental treatment, signs of respiratory infection, or gastrointestinal complaints. On physical examination, he did not appear acutely ill. The blood pressure was 130/93 mmHg, his pulse 120 beats per minute, and his auricular temperature 37.5°C. Except for a holosystolic heart murmur in the apical region, no abnormalities were found on examination. Specifically, no spinal percussion tenderness or focal neurological deficit was detected. His hemoglobin level was 11.0 g/dL (ref. 14–18 g/dL), and his white blood cell count was within the normal range. The erythrocyte sedimentation rate (46 mm/h; ref. 0–10 mm/h) and C-reactive protein (45 mg/L; ref <8 mg/L) were elevated. He received penicillin 12 *∗* 10^6^ U/24 h and gentamicin 3 mg/kg/24 h intravenously for possible endocarditis. Both transthoracic and transesophageal echocardiograms were performed, which showed mitral valve insufficiency based on a prolapse, but no vegetation or other echocardiographic signs of infectious endocarditis. Therefore, the Dukes criteria were not met, and endocarditis was ruled out with reasonable certainty. Gentamicin was discontinued, and the dose of penicillin lowered to 6 *∗* 10^6^ U/24 h. Lumbar MRI showed vertebral osteomyelitis at discus L2 and L3 (white arrow, Figure 1(a)). In total, our patient was treated with intravenous penicillin for *Granulicatella adiacens* osteomyelitis for three weeks, followed by two weeks of oral clindamycin (600 mg three times daily). Clindamycin was chosen for its high bone tissue penetration. Six weeks after cessation of antibiotic treatment, the patient had fully recovered. In addition, this was supported by low inflammation markers and negative follow-up blood cultures.

## 2. Results

Initial blood cultures (2 anaerobic and 2 aerobic bottles) were positive within 48 hours of withdrawal. Blood cultures were detected with BACTEC FX (Beckton Dickinson, model FTO-67) using standard blood culture bottles. Bacterial identification was performed using MALDI-TOF (Microflex LT MS, Bruker Daltonics). Follow-up blood cultures before start of antibiotic treatment, drawn two days after initial cultures, confirmed bacteremia with *Granulicatella adiacens* (Figures 1(b) and 1(c)). Antibiotic susceptibility was determined using broth microdilution and *e*-test and interpretation according to EUCAST breakpoint for streptococci species (Figure 1(d)).

## 3. Discussion

The abovementioned patient suffered from a rare case of *Granulicatella adiacens* vertebral osteomyelitis without adjacent involvement of a heart valve. The *Granulicatella* spp. were formerly known as nutritionally variant streptococci, which are now classified in two new genera, the *Abiotrophia* and *Granulicatella* [1]. They grow in Gram-positive cocci in pairs and chains; the optimal nutritional condition is pyridoxal-supplemented culture media [2]. The *G. adiacens* belongs to the common oral, genital, and intestinal flora in humans. This microorganism has been isolated in numerous infectious diseases, especially in the immune compromised host. Infectious endocarditis (IE) is the most reported infection due to *Granulicatella* spp. [3]. *Granulicatella* spp. are usually susceptible to penicillin, cephalosporins, and carbapenems. However, resistance to penicillin has been described and should be taken into account when considering empirical therapy [4]. To our knowledge, the literature describes only three cases of *Granulicatella* vertebral osteomyelitis without IE [5–7]. This is particularly exceptional because vertebral osteomyelitis is often the cause of hematogenous dissemination and therefore associated with endocarditis. Previous cases without IE as a primary focus describe patients with a healthy immune system, in which one was preceded by a dental procedure [5]. The present case is remarkable due to the low-grade infection, including bacteremia and vertebral osteomyelitis without involvement of the heart valves. However, in event of the presenting symptoms as mentioned in this case (fever and chills), a subacute endocarditis should always be considered. As mentioned above, the immunocompromised hosts are more susceptible to infection with *Granulicatella* spp. Nevertheless, our patient should not be considered as immunocompromised, since treatment with the immunosuppressant 6-mercaptopurine was stopped three months before the infection. Given the transient leukopenia, recovery of bone marrow function at the time of infection is presumable. Although no cultures were taken from the vertebra, MRI imaging combined with positive blood cultures are considered sufficient evidence for the current diagnosis. The diagnosis is confirmed by the multiple negative blood cultures after adequate antibiotic treatment.

## 4. Summary

In short, we report a rare case of *Granulicatella adiacens* vertebral osteomyelitis. In case of signs of back pain and low-grade infection without a clear focus, the practitioner should be aware of the possibility of an atypical infection with *Granulicatella* spp., even without additional signs of endocarditis.

## Data Availability

No data were used to support this study.

## Additional Points

In case of a vertebral osteomyelitis, the internist should be aware of the possibility of an infection with *Granulicatella* spp. *Granulicatella* spp. can be the cause of a low-grade bacteremia, not necessarily in the clinical presence of an infectious endocarditis.
